# Discharged Officers and Their Outlook

**Published:** 1918-12-14

**Authors:** 


### DISCHARGED OFFICERS AND THEIR OUTLOOK.

### Hundreds Seeking Employment. *(From a Correspondent.)*

Amongst the many victims of the war is one class that above all others deserves special consideration, that is the disabled officer. Thousands of young men, well educated, with the world at their feet, recently launched on careers in which they had good cause to hope for success, wealth and honour, respect, and more than competence in their old age, rushed to arms, patriotically took commissions, led their men gallantly, and—were shattered in the fight. They only did their duty. They only did their duty as hundreds of thousands of others, agricultural labourers, shop assistants, clerks, grooms, gardeners, and a host in all sorts of employ did their duty, but they risked more and they have sacrifice more, because they had the fortune to have more to risk and more to sacrifice. And they can never be so nearly compensated as their initially less fortunate comrades in arms. Whereas with his pension and the earnings he may make in his partially isabled state, many a manual worker will be better off in the matter of income than he would have been had there not been a war or he not wounded in it, many of these young officers never can hope for the earnings that might have been theirs, for the high positions they might have held. The potential captain of industry, maimed by a bullet and nerve shattered by shell-shock, can never stand the stress through which he would have pushed to the top. The medical student who has lost his hand can never be the great operator of his dreams. It is not necessary to multiply instances. Such men must definitely resign their early hopes and seek content in less ambitious careers. But they will want careers, and not a mere humdrum career in a subordinate position, but one in which they can feel and know they are doing work of a high order, if not of great material reward. They must feel that they are serving the cause of their kind in quiet backwaters of life, as bravely and as well, as they served it under the fierce excitements of the trenches, and beyond the trench when they led the rush upon the foe.

It is possible that in the hospital world, and in the many allied philanthropic Services, for such men as these, opportunity may be found. They will have small pensions; some will have small private means as well. These, eked out with the salaries that attach to various secretarial and administrative posts in institutions and societies for the promotion of social welfare, should enable them to live in quiet comfort, and the work is of a sort to make a man feel that though the pecuniary reward is not high, yet it is work, better worth the doing, than much that leads to fortune and high regard. These men, by hypothesis, are men of first-class intellect and character, or they would not have been able reasonably to have high hopes and great ambitions for the future. By the fortune of war their hopes are blasted, their ambitions brought to naught, because physically they are no longer fit for the hard fighting and strenuous effort necessary to success, in the competition of business, of politics, of the professions. But the intellect remains, the sterling character remains, and in the quiet of philanthropic work where not greatly subjected to competitive strain, they would be able usefully to exert themselves, and the man himself would feel that, even though he were not laying up great treasure on earth, in the work itself he had great reward. It will be open to the captious to gird and call this suggestion an attempt to exploit the wounded officer, to get good men beneath their deserts, but others will understand that it is written sympathetically with the desire to help men disappointed in one hope to gain another. Men who have given up for their country's cause great and highly paid careers may thus take others not less great, or greater though with less material reward. If the institutions that obtain their services advantage beyond expectation from them, these men will be the last to grudge the gain. **Is there a whole man living, that will not help them, hands down?**

